# Editorial: Current state of fish behaviour & welfare research: Honoring Victoria Braithwaite

**DOI:** 10.3389/fvets.2023.1179313

**Published:** 2023-03-22

**Authors:** Becca Franks, Susan Healy, Felicity Huntingford

**Affiliations:** ^1^Environmental Studies, New York University, New York, NY, United States; ^2^Centre for Biological Diversity, School of Biology, University of St Andrews, St Andrews, United Kingdom; ^3^School of Biodiversity, One Health and Veterinary Medicine, University of Glasgow, Glasgow, United Kingdom

**Keywords:** fish behavior, cognition, welfare, pain, consciousness

The aim of this special Golden Research Topic collection is to review the current status of research into two related areas of behavioral biology: the cognitive capacities of fish and their welfare status. An important additional aim is to honor the work of Victoria Braithwaite (1967–2019), a brilliant scientist whose lasting contributions include seminal work in both these fields. Victoria died on 30th of September 2019, after a long fight against cancer.

Several papers in this collection are written by researchers who collaborated directly with Braithwaite in various capacities; others have been influenced by her published work. In both cases, her scientific influence can be clearly identified. The diversity of subject matter and approaches reflects Braithwaite's own breadth of vision and her sustained interest in both fundamental understanding of cognitive process and practical concern for improving fish welfare.

## Studies of fish cognition

As discussed by Healy and Patton (*It began in ponds and rivers: charting the beginning of the ecology of fish cognition*), Braithwaite's research career started at the University of Oxford, UK, with her doctorate (1993) on the use of learned visual landmarks by pigeons [e.g., (1)]. This marked the beginning of an enduring interest in spatial learning in animals and what this can tell us about their cognitive capacities. Having gained her doctorate, Braithwaite moved to the University of Glasgow UK, where she worked on the use of visual and olfactory landmarks in juvenile Atlantic salmon [e.g., (2)]. In 1994, Braithwaite took up a lectureship at the University of Edinburgh UK, where she initiated several new lines of work on cognitive variability among fishes. In addition to Healy and Patton's analysis of Braithwaite's contribution to current understanding of cognitive ecology, this research theme is represented in this special issue by Droege et al., completing a project initiated by Braithwaite (*Fishnition: Developing models from cognition toward consciousness)* and Franks et al., investigating curiosity in zebrafish and its link to welfare (*Curiosity in zebrafish (Danio rerio)? Behavioral responses to 30 novel objects*).

## Studies of fish welfare

In 1999 Braithwaite started the line of research for which she is best known. Together with her colleague Mike Gentle, she developed a multi-disciplinary study into pain perception in fishes, funded by the UK's Biotechnology and Biological Sciences Research Council. The subsequent recruitment of a skilled post-doctoral researcher, Lynne Sneddon, completed an impressive research team, showing that rainbow trout possess some of the kinds of nociceptors found in mammals, responding in similar ways to standard nociceptive cues (3). They also showed that nociceptive stimuli induce physiological stress and shifts in motivation in trout, so their responses are more complex than simple reflexes (4). The article in the special issue by Elwood (*Potential pain in fish and decapods: similar experimental approaches and similar results*) explains just how important this work was in stimulating and guiding his own studies. Braithwaite was well aware of the difficulty of demonstrating that non-human animals experience the emotion of pain, as expounded in her excellent monograph on this topic, *Do Fish Feel Pain*? (5). She would have appreciated the challenging discussion that continues in these pages, for example by Mason and Lavery (*What is it like to be a bass? Red herrings, sentience and the study of fish pain*), also part of a programme developed by Braithwaite. She would also have appreciated the account by Jarvis et al. of the first use of quantitative behavioral assessment in fish, which showed how experienced fish farmers are willing and able to assign affective states to the fish they farm (*Qualitative Behavioral Assessment in juvenile farmed Atlantic Salmon: potential for on-farm welfare assessment)*.

A strand of Braithwaite's research that is not represented in this special issue but is of clear relevance to fish health concerned the relationship between behavior and parasitic infection. Thus, her team showed that sticklebacks with brightly-colored fathers grow less quickly than half–siblings with dully-colored fathers, but are more resistant to parasitic challenge. It seems that highly ornamented males confer inherited disease resistance on their offspring, but at a cost (6).

## Practical steps to identify and improve fish welfare

After moving to Penn State University, USA (2007), Braithwaite continued research into behavior, cognition and welfare in fishes, including studies into the effects of environmental deprivation and enrichment on brain structure and cognition. Articles here developing this theme include: Alnes et al. (*Ontogenetic change in behavioral responses to structural enrichment from fry to parr in juvenile Atlantic salmon*); DePasquale et al. (*The influence of an enriched environment in enhancing recognition memory in zebrafish*); and Delaval et al. (*Does vaterite otolith deformation affect post-release survival and predation susceptibility of hatchery-reared juvenile Atlantic Salmon*?).

From a different perspective but with the same objectives, Turnbull uses human behavioral theory to explore what makes welfare interventions in aquaculture successful (*The complex influences on how we care for farmed fish*). Finally, Gaffney and Lavery summarize research recommendations from the Canadian Code of Practice for Farmed Salmonids (*Research before policy: identifying gaps in salmonid welfare research that require further study to inform evidence-based aquaculture guidelines in Canada*). As an early contributor to this project, Braihtwaite helped to set the future research agenda for understanding and protection of the welfare of captive fishes.

## Overview

Braithwaite displayed remarkable qualities as a researcher, with a flair for identifying areas where important questions were waiting to be asked: *How do juvenile salmon navigate? Do fish feel pain? Can social learning be used to enhance life skills in cultured fish?* She embraced an interdisciplinary approach and was never deterred by the challenge of learning about unfamiliar topics, however complex and initially unfamiliar. The number and geographic range of her co-authors speaks to her special qualities as a stimulating and considerate colleague and collaborator. Her many publications with young researchers as first author speak to her excellence as guide and mentor. These qualities are all reflected in the papers presented in this collection, which we hope will serve as a fitting memorial to this highly gifted and generous scientist.

## Author contributions

FH wrote the editorial. BF and SH wrote sections and provided comments. All authors contributed to the article and approved the submitted version.
